# p53 Regulates Oxidative Stress-Mediated Retrograde Signaling: A Novel Mechanism for Chemotherapy-Induced Cardiac Injury

**DOI:** 10.1371/journal.pone.0018005

**Published:** 2011-03-30

**Authors:** Joyce M. Velez, Sumitra Miriyala, Ramaneeya Nithipongvanitch, Teresa Noel, Chotiros D. Plabplueng, Terry Oberley, Paiboon Jungsuwadee, Holly Van Remmen, Mary Vore, Daret K. St. Clair

**Affiliations:** 1 Graduate Center for Toxicology, University of Kentucky, Lexington, Kentucky, United States of America; 2 Department of Pathology and Laboratory Medicine, University of Wisconsin, Madison, Wisconsin, United States of America; 3 Pathology and Laboratory Medicine Service, William S. Middleton Memorial Veterans Administration Hospital, Madison, Wisconsin, United States of America; 4 The Faculty of Medical Technology, Mahidol University, Bangkok, Thailand; 5 Department of Cellular and Structural Biology, University of Texas Health Science Center at San Antonio, San Antonio, Texas, United States of America; Roswell Park Cancer Institute, United States of America

## Abstract

The side effects of cancer therapy on normal tissues limit the success of therapy. Generation of reactive oxygen species (ROS) has been implicated for numerous chemotherapeutic agents including doxorubicin (DOX), a potent cancer chemotherapeutic drug. The production of ROS by DOX has been linked to DNA damage, nuclear translocation of p53, and mitochondrial injury; however, the causal relationship and molecular mechanisms underlying these events are unknown. The present study used wild-type (WT) and p53 homozygous knock-out (p53^−/−^) mice to investigate the role of p53 in the crosstalk between mitochondria and nucleus. Injecting mice with DOX (20 mg/kg) causes oxidative stress in cardiac tissue as demonstrated by immunogold analysis of the levels of 4-hydroxy-2′-nonenal (4HNE)-adducted protein, a lipid peroxidation product bound to proteins. 4HNE levels increased in both nuclei and mitochondria of WT DOX-treated mice but only in nuclei of DOX-treated p53^(−/−)^ mice, implicating a critical role for p53 in causing DOX-induced oxidative stress in mitochondria. The stress-activated protein c-Jun amino-terminal kinase (JNKs) was activated in response to increased 4HNE in WT mice but not p53^(−/−)^ mice receiving DOX treatment, as determined by co-immunoprecipitation of HNE and pJNK. The activation of JNK in DOX treated WT mice was accompanied by Bcl-2 dissociation from Beclin in mitochondria and induction of type II cell death (autophagic cell death), as evidenced by an increase in LC3-I/LC-3-II ratio and γ-H2AX, a biomarker for DNA damage. The absence of p53 significantly reduces mitochondrial injury, assessed by quantitative morphology, and decline in cardiac function, assessed by left ventricular ejection fraction and fraction shortening. These results demonstrate that p53 plays a critical role in DOX-induced cardiac toxicity, in part, by the induction of oxidative stress mediated retrograde signaling.

## Introduction

Research on mitochondria has evolved from emphasizing bioenergetics to studying biogenesis, the genetic functions of mitochondrial DNA (mtDNA), and diseases associated with mitochondrial dysfunction. Although these areas continue to be investigated vigorously, a new era in mitochondrial research has emerged that concerns the role of this organelle in intracellular signaling.

p53, an important tumor suppressor gene, is recognized as the guardian of the genome because it regulates the transcription of numerous genes that code for life and death processes. However, during the last decade, the transcription-independent activity of the p53 protein has emerged as an important mechanism by which p53 modulates mitochondrial function. p53 interacts with various proteins in the outer membrane as well as in the matrix of the mitochondria, including bcl-2-associated X protein (Bax), Bcl_2_, p53 up-regulated modulator of apoptosis (PUMA) [1], polymerase gamma [2], and manganese superoxide dismutase (MnSOD) [3]

It is well documented that free radical-mediated oxidative stress plays a pivotal role in the cardiac toxicity of Doxorubicin (DOX) [4]. We have shown that overexpression of human MnSOD, a primary antioxidant enzyme located in the mitochondrial matrix, protects against DOX-induced cardiac injury, suggesting that the DOX-induced cardiac injury is related to the effect of DOX on cardiac mitochondria [4]. However, the pathways that mediate the observed protective effect of MnSOD remain unknown.

ROS are highly reactive and, when generated close to cell membranes, oxidize membrane phospholipids (lipid peroxidation), which can lead to the generation and accumulation of lipid peroxidation products, such as malondialdehyde, 4-hydroxy-2-nonenal (4HNE), acrolein and F2-isoprostanes. 4HNE is a highly reactive and specific diffusible end-product of lipid peroxidation and is known to induce/regulate various cellular events such as proliferation and growth inhibition [5], T cell apoptosis [6] and activation of signaling pathways [7]. Proteins are major targets of 4HNE, which can trigger multiple modifications of the protein structure. 4HNE has a high affinity towards cysteine, histidine and lysine residues forming direct protein-adducts and thereby altering protein function.

Autophagy (Greek: ‘to eat oneself’) is an intracellular event in which a cell digests its own constituents. The term “autophagic cell death” describes a form of programmed cell death morphologically distinct from apoptosis and presumed to result from excessive levels of cellular autophagy [8]. In classical apoptosis, or type I programmed cell death, there is early collapse of cytoskeletal elements but preservation of organelles until late in the process. In contrast, in autophagic, or type II, programmed cell death there is early degradation of organelles but preservation of cytoskeletal elements until late stages.

Recent studies have demonstrated interactions between the autophagic and apoptotic pathways. The Bcl-2 family has been implicated in the crosstalk between apoptosis and autophagy [9]. Other apoptosis-related proteins such as p53 have also been shown to play a role in autophagy [10]. Beclin1, a mammalian autophagy gene, was originally identified as a bcl-2-interacting protein [11]. Members of the beclin1 and Bcl-2 family serve as a point of crosstalk between the autophagic and apoptotic pathways. Beclin1 directly interacts not only with bcl-2 but also with other anti-apoptotic proteins of the bcl-2 family such as bcl-xL [9]. Cardiac bcl-2 overexpression also inhibits autophagy in murine heart cells [12].

This present study focuses on an understanding of the molecular mechanisms underlying DOX-induced cardiac toxicity. It identifies p53 as a candidate in augmenting retrograde signaling leading to cardiac injury by using genetic manipulations, functional tests, ultrastructural analyses, and biochemical assays.

## Results

### Absence of p53 selectively reduces DOX-induced oxidative damage in the mitochondria

ROS are so reactive that they exist in tissues for only short periods of time, which makes measuring them difficult. One approach to solving this problem is to rely on biochemical assays of oxidatively damaged products. A major problem with these assays, however, is that the oxidatively damaged products are susceptible to further in vitro oxidation, which hinders accurate measurement of levels of oxidatively damaged products in cellular organelles and in specific cell types from complex tissues. To circumvent this problem, the Oberley [13] laboratory and others have developed techniques to localize and quantify oxidatively damaged products using specific antibodies coupled with electron microscopy procedures. Two previous studies have validated the immunomorphologic approaches presented herein by documenting good agreement between biochemical and immunomorphologic analyses in an experimental model of oxidative stress (iron nitrilotriacetate-induced injury of rat kidney [14] and analysis of oxidative damage in skeletal muscle of aging rhesus monkey [15]).

We have previously shown that DOX causes significant oxidative damage manifested by increased levels of 4HNE adducted protein levels in mitochondria [16], [17]. Recent studies suggest that p53 plays an important role in maintaining mitochondrial function, but it is not known whether the oxidative stress status of the nucleus and mitochondria are differentially affected by p53.

**Figure 1:**
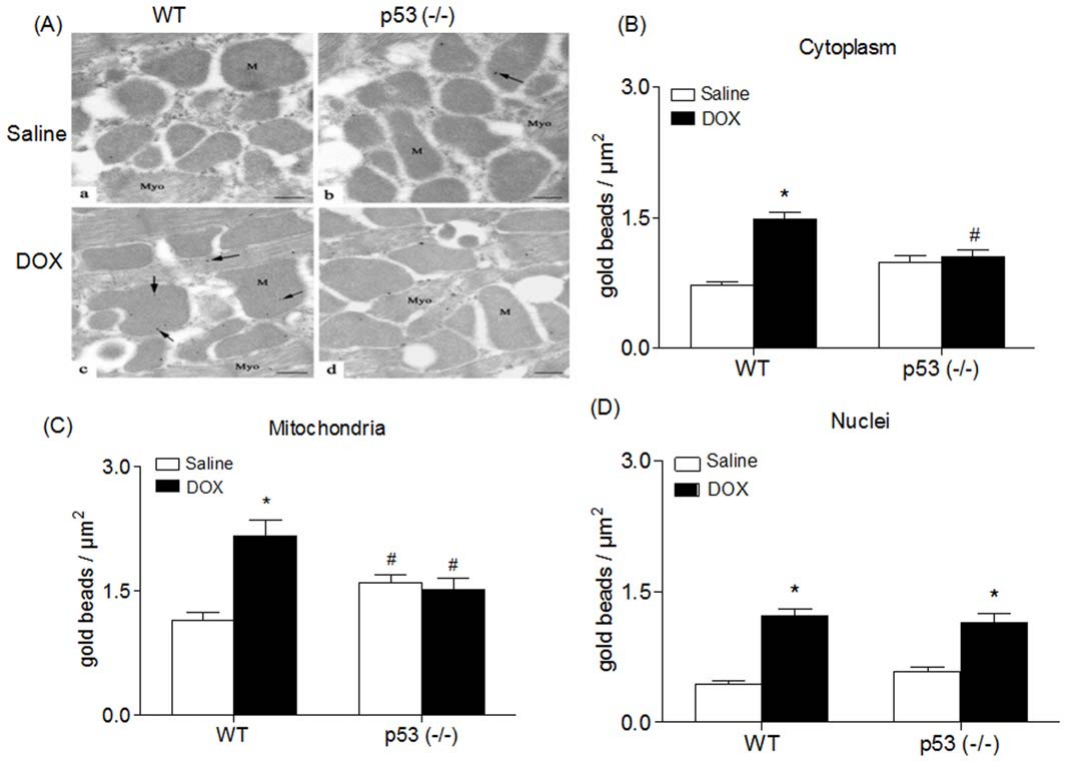
Absence of p53 selectively reduces DOX-induced oxidative damage in the mitochondria. A. Representative immunogold electron micrographs using antibody against 4HNE protein adducts in cardiomyocytes. Electron dense beads indicate positive staining for 4HNE protein adducts (arrow). Left ventricular tissues from WT (a) and p53^(−/−)^ (b) mice treated with saline demonstrate low labeling of 4HNE in mitochondria (M) and myofilaments (Myo) (a and b). Significant increase in labeling of 4HNE protein adducts was observed in both mitochondria and myofilaments of the WT mice hearts (c) but not in the p53^(−/−)^ mice (d) treated with DOX. Scale bar, 1 µm. B, C, and D. 4HNE-immunoreactive protein adducts were quantified in cardiomyocyte cytoplasm (B), mitochondria (C) and nuclei (D) for both WT and p53^(−/−)^ mice treated with saline and DOX. Labeling density is expressed in gold beads/µm^2^. All graphs represent the Mean ± SEM for each group. *p<0.05 when compared with saline treated mice of the same genotype, ^#^ p<0.05 when compared with WT mice similarly treated.

To directly address this issue, we examined the levels of 4HNE-protein adducts in cardiomyocytes of WT and p53^(−/−)^ mice using quantitative ultrastructural immunogold analysis techniques. As illustrated in Figure 1A, gold labeling of 4HNE-protein adducts was infrequently found in cardiomyocyte mitochondria and cytoplasm of saline treated WT and p53^(−/−)^ mice (Figure 1A, subpanel a and b). An increase in 4HNE-modified proteins labeling was observed in mitochondria of cardiomyocytes from DOX-treated WT but not p53^(−/−)^ mice (Figure 1A, subpanel c and d). Quantification of 4HNE immunoreactive protein adducts in cardiomyocytes of DOX treated WT mice demonstrated a significant increase in mitochondria (Figure 1C), cytoplasm (Figure 1B) and nuclei (Figure 1D) in comparison with saline controls. A significant increase was also observed in the nuclei (Figure 1D) but not in cytoplasm (Figure 1C) or mitochondria (Figure 1D) of DOX-treated p53^(−/−)^ mice in comparison with saline treated controls. Interestingly, cardiomyocytes from saline-treated p53^(−/−)^ mice exhibited higher levels of 4HNE-protein adducts in the mitochondria in comparison with saline-treated WT mice (Figure 1C) while levels in cytoplasm (Figure 1B) and mitochondria (Figure 1C) from DOX-treated p53^(−/−)^ mice were significantly lower than those in WT mice similarly treated. These results confirm our previous observations regarding the effect of DOX on mitochondrial 4HNE levels [18]. The results demonstrate that p53 plays a major role in increasing mitochondrial 4HNE after DOX treatment.

### 4HNE induces JNK activation and Bcl-2 phosphorylation, and triggers autophagic cell death

**Figure 2:**
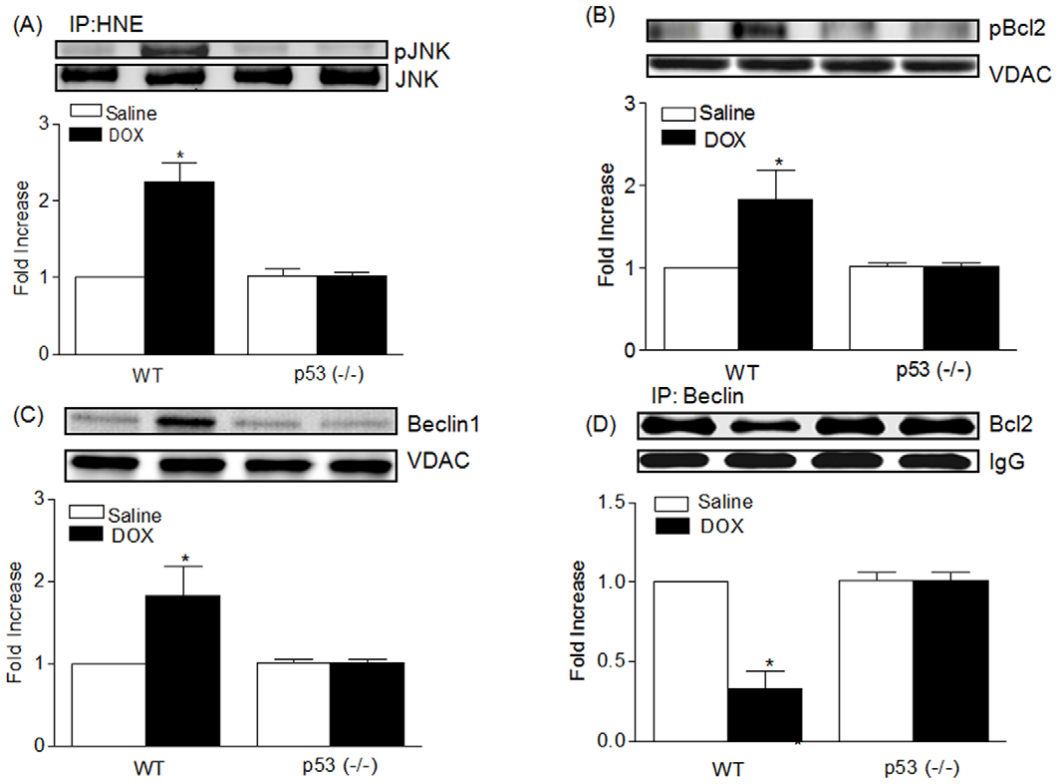
DOX induces HNE adduction with pJNK and Bcl-2 phosphorylation, and triggers autophagic response. A. PhosphoJNK was detected by immunoprecipitation using a polyclonal goat anti-HNE antibody followed by Western blot analysis with a rabbit polyclonal Thr183/Tyr185 phosphorylation specific JNK antibody. Quantitative analysis represents the Mean ± SEM (n = 5) in each group; *p<0.001 as compared to other groups. B. Western blot analysis of p-Bcl2 in cardiac mitochondria from WT and p53^(−/−)^ mice treated with saline or DOX and quantitative analysis represents the Mean ± SEM n = 5 in each group; *p<0.001 as compared to other groups. C. Western blot analysis of Beclin1 in cardiac mitochondria from WT and p53^(−/−)^ mice treated with saline or DOX and quantitative analysis represents the Mean ± SEM n = 5 in each group; *p<0.001 as compared to other groups. D. Co-immunoprecipitation of Beclin1 with Bcl-2 in cardiac mitochondria from WT and p53^(−/−)^ mice treated with saline or DOX and quantitative analysis represents the Mean ± SEM n = 5 in each group; *p<0.01 as compared to other groups.

JNKs are involved in activation of caspases [19], [20], but their role is not uncomplicated. JNK activation could be either upstream [20] or downstream [19] of cell death activation, depending on cell type and death-initiating agents. We therefore evaluated whether DOX treatment increases JNK in mitochondria as a result of 4-HNE increase, using a phospho-specific antibody that recognizes dual site phosphorylation of JNK at Thr183 and Tyr185 (Figure 2A). While the total levels of JNK were equivalent in both genotypes under identical treatment conditions, little phosphorylated JNK was detected in p53^(−/−)^ mice treated with DOX. In contrast, a high level of phosphorylated JNK was detected in WT mice treated with DOX. The WT but not p53^(−/−)^ mice treated with DOX exhibited increased levels of Bcl-2 phosphorylation (Figure 2B) and its associated protein beclin1 (Figure 2C). Beclin1-Bcl-2 co-immunoprecipitation results suggest that the increased phosphorylation of Bcl-2 inhibits interaction with Beclin1, which is most evident in WT mice treated with DOX (Figure 2D).

**Figure 3:**
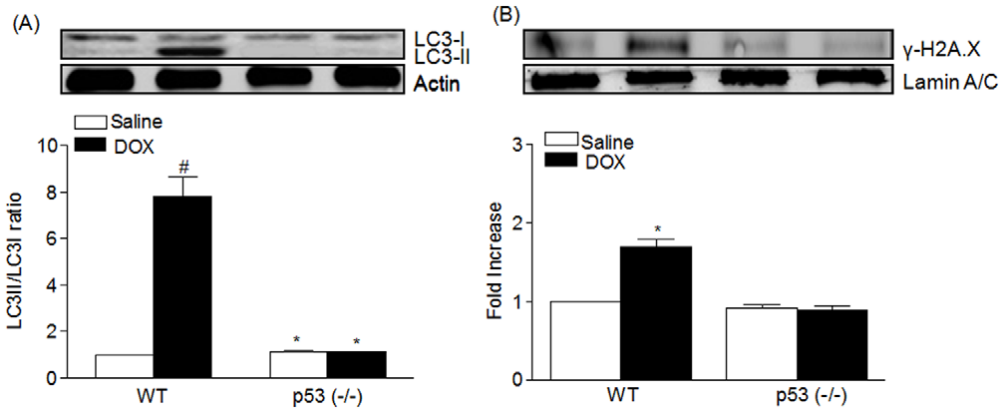
p53 triggers autophagy and cell death. A. Biochemical marker for cardiac injury by autophagy. Western blot analysis of autophagy marker (LC3) expression in heart tissue homogenates from WT and p53^(−/−)^ mice treated with saline or DOX. Quantitative analysis represents the Mean ± SEM n = 5 in each group; ^#^p<0.001 as compared to other groups. B. Western blot analysis of gamma H2AX marker for cell death in heart tissue nuclear extracts from WT and p53^(−/−)^ mice treated with saline or DOX. Quantitative analysis represents the Mean ± SEM n = 5 in each group. ^#^p<0.001 as compared to other groups.

Activation of the JNK pathway has been associated with induction of type II death (autophagic cell death). To verify the role of p53 in DOX-induced autophagic cell death at a biochemical level, the conversion of LC3-I to LC3-II, an indicator of autophagy activation, was assessed by immunoblot analysis using LC3 antibody (Figure 3A). LC3-II cleavage product was significantly increased (p<0.001) in DOX-treated WT compared to the p53^(−/−)^ mice similarly treated. The role of p53 in type II death was assessed also by γ-H2AX as a biomarker for DNA damage (Figure 3B),in DOX-treated WT, but not in p53^(−/−)^ mice.

### p53 contributes to DOX-induced cardiac injury

**Figure 4:**
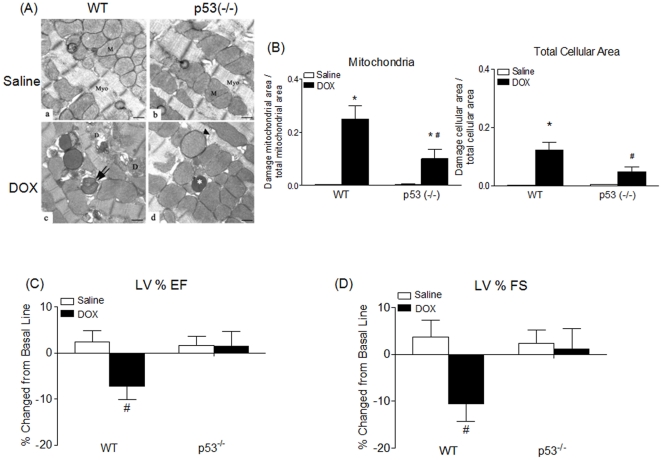
p53 contributes to DOX-induced cardiac injury. A. Representative electron micrographs demonstrating cardiomyocyte injury and morphometric quantification of subcellular injury in cardiomyocytes in WT and p53^(−/−)^ mice. WT (a) and p53^(−/−)^ (b) mice treated with saline demonstrated normal ultrastructure of heart muscle with cardiomyocytes showing numerous mitochondria (M), prominent myofilaments (Myo) and lipids (Lip). WT (c) and p53^(−/−)^ (d) mice treated with DOX demonstrated the following pathologic changes: lysosomal degradation of mitochondria (asterisk), mitochondria with loss of cristae (arrow), peri-mitochondrial swelling (double arrow), disruption of mitochondrial membranes (arrowhead), and mitochondrial degeneration (d); scale bar, 1 µm. B. Pathologic changes were quantified for saline and DOX treated mice in the mitochondria and total cellular area (excluding nuclei). Quantification of damage for each specific compartment is expressed in the ratio of damaged area versus the total area. All graphs represent the Mean ± SEM for each group. *p<0.05 when compared with saline treated mice of the same genotype, and ^#^p<0.05 when compared with WT mice treated with DOX. n = 6 or 7. C & D. Left ventricular function, assessed by percentage of ejection fraction (LV%EF) (C) and fractional shortening (LV%FS) (D), is expressed in percentage of change from basal levels caused by saline and DOX three days after injections for WT and p53^(−/−)^ mice. All bar graphs represent the Mean ± SEM for each group. ^#^p<0.05 when compared to all three other groups. n = 6–11.

To verify that the observed changes in biochemical markers are accompanied by cardiac injury, we examined the ultrastructural morphology of cardiac tissues, including mitochondrial injury associated with lysosomal degradation of mitochondria, loss of cristae, degeneration of mitochondria, peri-mitochondrial swelling and disruption of mitochondrial membranes (Figure 4A). Quantification of subcellular injury demonstrated a significant increase of cardiomyocyte mitochondria injury in both WT and p53^(−/−)^ mice hearts following DOX treatment (Figure 4B). However, the levels of total cellular and mitochondrial damage in WT cardiomyocytes were significantly greater than in the p53^(−/−)^ cardiomyocytes similarly treated (p<0.05) (Figure 4B). Thus, the results from ultrastructural analysis suggest that p53 plays a role in exacerbating DOX-induced mitochondrial injury in cardiac tissues.

Echocardiography detects a decline in cardiac function in patients who have received an infusion of DOX [21] as part of chemotherapy. We and others have demonstrated that three to five days after an injection of DOX, cardiac function significantly declines in WT mice [22], [23]. WT mice treated with DOX showed a significant decrease (p<0.05) in the LV%EF (Figure 4C) and LV%FS (Figure 4D), but the p53^(−/−)^ mice similarly treated did not. These results demonstrate that the lack of biochemical changes in p53 deficient mice is accompanied by a significant reduction of DOX-induced structural damage and cardiac dysfunction.

## Discussion

p53 is regarded as the guardian of the genome with the capability of regulating many life and death processes, but whether it plays a role in regulating retrograde signaling is unexplored. The best known function of p53 is its action as a transcription factor. p53 can activate or suppress expression of its target genes in a number of ways. The typical targets of p53, such as GADD45, p21, PUMA, BAX, and SCO2, are involved in DNA repair, cell cycle progression, metabolism and apoptosis. Under physiological conditions, cellular p53 is kept at a low level by an autoregulatory loop with MDM2, a p53 target that mediates p53 ubiquitination and subsequent degradation by the 26S proteosome [24]. Upon exposure to various forms of stress, such as DNA damage, oncogenic activation, hypoxia, mitotic apparatus dysfunction, telomere erosion, energy stress or oxidative stress, p53 coordinates cellular responses through both transcription-dependent and -independent mechanisms. These responses prevent or repair genomic damage, or eliminate cells when damage is beyond repair.

The present study identifies a novel mechanism by which p53 activation selectively enhances DOX-induced oxidative stress in mitochondria, which is manifested by an increase in the level of 4HNE-adducted proteins in the mitochondria. Under physiological conditions, 4-HNE may act as a signaling molecule to maintain normal cellular functions [25]. Because of the reactivity of this and other aliphatic and aromatic aldehydes, cells have developed mechanisms to detoxify these molecules [25]. Accumulation of 4HNE, which can induce cellular injury, may be caused by deficiencies in the process of toxic product elimination. Oxidative stress increases HNE-adducted proteins. This increase is partially reduced by the conjugation of HNE with GSH that forms the glutathione conjugate of HNE (GS-HNE) [26], which is a substrate of the multidrug resistant associated protein, MRP1 [27]. Perfusion of the rat heart with HNE leads to the formation and efflux of GS-HNE [28], suggesting a role for MRP1 in the clearance of GS-HNE from cardiac tissue. We have previously observed that MRP1 likely mediates the saturable efflux of the glutathione conjugate of HNE observed upon infusion of HNE to the perfused heart [29] that protects the cardiomyocytes. However, under conditions of oxidative stress, accumulated 4HNE modifies and regulates enzymes involved in mitochondrial energy production [30], which results in the increased ROS generation [31] and diminished protein degradation [32] that may activate autophagic pathways.

The JNK1 signaling pathway has been shown to regulate autophagy in both Drosophila and mammalian cells in response to not only starvation but also ER stress and cytokine stimulation (e.g., IL-2, TNFα) [33]. The diversity of stress stimuli capable of triggering JNK1-mediated autophagy is consistent with the hypothesis that autophagy regulation is intricately intertwined with numerous other cellular stress response programs mediated by JNK1 signaling. Despite the known link between JNK1 and autophagy induction, the mechanism by which JNK1 activation leads to autophagy induction remains undefined. In the present study, we show that activation of JNK by 4HNE is rapid and sustained suggesting that sustained activation and phosphorylation of JNK may be needed for 4HNE-induced type II programmed cell death. The JNK1 signaling pathway is associated with Bcl-2 phosphorylation, which leads to disruption of the Bcl-2/Beclin1 complex and activation of Bcl-2 inhibition of Beclin1-dependent autophagy. Our finding shows that the level of phospho-Bcl-2 is increased while the level of Bcl-2 is reduced in DOX-treated p53 wild-type mice which are consistent with the p53-mediated increase in activated JNK1 in DOX-treated mice. This possibility is further confirmed by the finding that in p53^(−/−)^ mice, there is no increase in JNK1 activation or Bcl-2 phosphorylation.

It is possible that autophagy may have a protective role in tissue remodeling [10], However, our results suggest that autophagy induced by DOX treatment is not protective to cardiac tissues because the absence of autophagy in DOX-treated p53^(−/−)^ mice is accompanied by the reduced cardiac injury observed morphologically and functionally.

**Figure 5:**
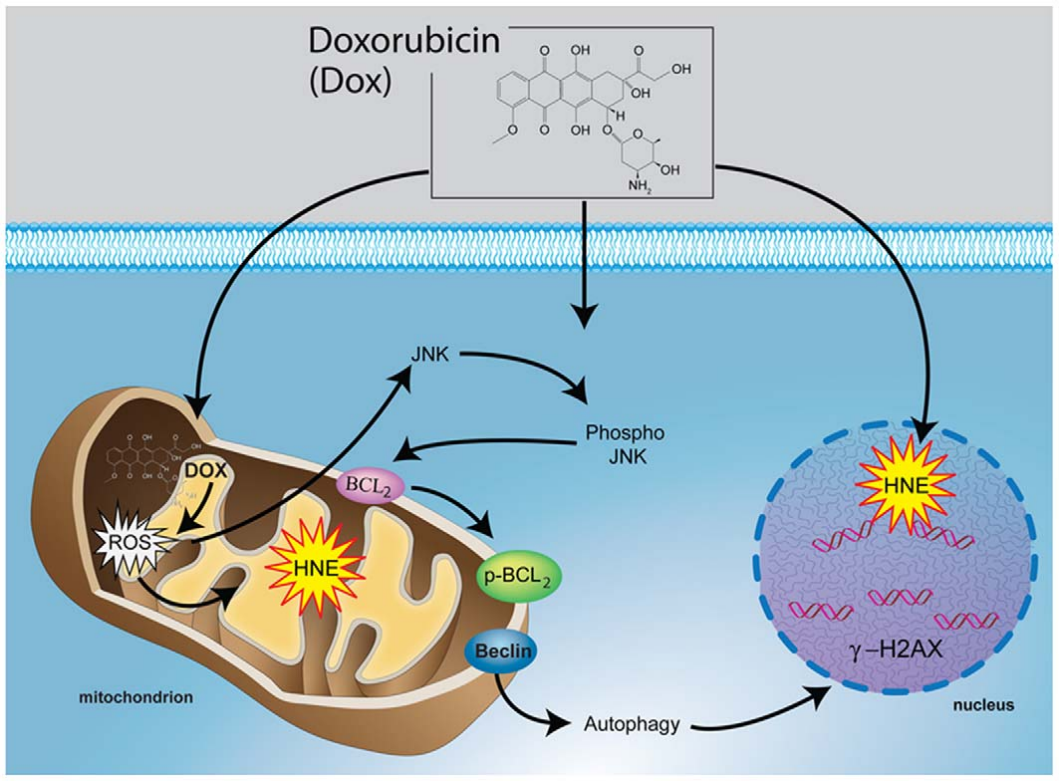
p53 enhances DOX-induced cardiac injury, in part, via enhancement of oxidative stress in mitochondria. The absence of p53 selectively prevents DOX-mediated increase in oxidative stress indicators, including 4HNE-adducted proteins in mitochondria but not in the nucleus. DOX-induced p53-dependent increased oxidative stress in mitochondria is associated with sustained activation of JNK1 and subsequent phosphorylation of Bcl-2 and release of beclin from the Bcl-2-beclin complex resulting in detrimental effect of autophagy (cellular injury).

In conclusion, the present study demonstrates that treatment with DOX causes oxidative stress in cardiac tissue as evidenced by immunogold staining of 4HNE-adducted protein. The 4HNE levels were increased in both nuclei and mitochondria of WT DOX-treated mice but only in nuclei of DOX-treated p53^(−/−)^ mice. JNKs were activated in response to increased 4HNE in WT mice but not in p53^(−/−)^ mice after DOX treatment. The activation of the JNK pathway by 4HNE was accompanied by induction of autophagic cell death as indicated by an increase in LC3-I/LC-3-II ratio and γ-H2AX in DOX treated WT mice (Figure 5). The absence of p53 significantly reduced mitochondria injury and DOX-induced cardiac toxicity, suggesting that p53 plays a critical role in mediating DOX-induced cardiac toxicity, in part, by the induction of oxidative stress mediated retrograde signaling.

## Materials and Methods

### Animals

Heterozygous mice (p53^(−/+)^) were maintained in our laboratory to produce p53^(−/−)^ and wild-type (WT) littermates. p53^(−/−)^ mice are in the C57BL/6 background and were initially generated in the laboratory of Dr.Tyler Jacks at the Center for Cancer Research and Department of Biology, Massachusetts Institute of Technology, Cambridge, MA. The targeted disrupted p53 genes do not produce p53 protein, since 40% of their gene coding region is eliminated by the induced mutation [34]. Male mice between 10 and 12 weeks old were used in all studies. All animal experimental procedures were approved by the Institutional Animal Care and Use Committee of the University of Kentucky.

### DOXorubicin treatment and tissue collection

Mice were treated with a single dose of 20 mg/kg of DOXorubicin-Adriamycin (DOXOrubicin HCl, from Bedford Laboratories, Inc., Bedford, OH) (DOX) or saline via intraperitoneal injection (IP). Three days after treatment, mice were anesthetized using Nembutal\sodium solution (65 mg/kg) (Abbott Laboratories, North Chicago, IL). The heart was excised and immediately processed for ultrastructural studies or frozen in liquid nitrogen for molecular and biochemical studies.

### Immunogold detection of 4HNE

Samples for staining were obtained from the same tissues used for ultrastructural examination (described above). Procedures to detect 4HNE were followed as previously described by Oberley *et al.* [13] and Nithipongvanitch *et al.* [35].

### Mitochondrial extract preparation

Heart mitochondria were isolated as described previously by Mela and Seitz [36] and [37]. Briefly, the hearts were collected, rinsed in ice-cold isolation buffer (0.225 M mannitol, 0.075 M sucrose, 1 mM EGTA, pH 7.4), and cut into small pieces. The heart tissue was washed three times with the isolation buffer to remove any residual blood and homogenized at 500 rpm with a chilled Teflon pestle in a glass cylinder with 10 strokes. The homogenate was centrifuged at 480 g at 4°C for 5 min in a Sorval SS 34 rotor. The resulting supernatant was filtered through a double-layered cheesecloth and centrifuged at 7700 g at 4°C for 10 min. Supernatant was saved to check for leakage from mitochondria using MnSOD, a mitochondrial matrix enzyme. The pellet was rinsed with 0.5 mL of the isolation buffer with gentle shaking to remove the “fluffy layer” (damaged mitochondria) on top of the pellet. The wall of the centrifuge tube was cleaned with cotton swabs to remove lipids. The pellet was washed by gentle resuspension in 3 mL isolation buffer using the smooth surface of a glass rod and centrifuged at 7700 g at 4°C for 10 min. The supernatant was saved to check again for leakage from the mitochondria. The washing was repeated once. The resulting mitochondria were collected for further analysis. The purity of mitochondria was examined using Lamin A (nuclear protein) and IĸB-α (cytoskeletal protein) by Western blotting. Protein content in the lysate was determined by BCA protein assay (Pierce, Rockford, IL).

### Immunoprecipitation and immunoblotting

Frozen hearts were homogenized with lysis buffer (10 mM Tris–HCl pH 7.2, 1%Nonidet P-40, 158 mM NaCl, 1 mM EDTA, 50 mM NaF, 1 mM PMSF, 10 µg/ml aprotinin, 10 µg/ml leupeptin, 1 mM sodium orthovanadate, 10 mM sodium pyrophosphate) and lysates centrifuged at 16,000×g for 10 min. Immunoblotting (40 µg protein/well) was performed according to Odyssey infrared imaging system (LI-COR, Lincoln, NE) with antibodies against LC3, Beclin1, γ-H2Ax and Bcl-2 (Cell Signaling Technology, Danvers, MA) and actin (Santa Cruz Biotechnology, Santa Cruz, CA) as the loading control. Secondary antibodies were conjugated with Alexa680 (Molecular Probes, Carlsbad, CA) or IRdye800 (Rockland Immunochemicals, Gilbertsville, PA), and detected and quantified using the Odyssey infrared imaging system (LI-COR, Lincoln, NE).

For immunoprecipitation, 250 µg of protein was taken and antibodies (2.5 µg/ml) were incubated for 2 h at 4°C with protein G-conjugated agarose beads (30 µl) and then washed five times with lysis buffer. To immunoprecipitate endogenous HNE and endogenous p-JNK, anti-HNE immunoprecipitates were subjected to SDS-PAGE and phospho-JNK (pJNK; Thr^183^/Try^185^) (Santa Cruz Biotechnology, Santa Cruz, CA) was detected by immunoblot. Similarly for Beclin1 and endogenous Bcl-2, anti-Beclin1 immunoprecipitates were subjected to SDS-PAGE, and Bcl-2 was detected by immunoblot analysis.

### Ultrastructural examination of cardiac tissues

Left ventricle tissues were processed for electron microscopic analysis followed by quantification of mitochondria, cytoplasm, and total cellular area damage using strict criteria for each compartment injury and image analysis techniques as previously described by Chaiswing *et al.* [18].

### Cardiac function assessment

Echocardiography measurements were taken at three days after a single IP injection with 20 mg/kg DOX or saline with a high-frequency 30 MHz probe (Vevo 660, VisualSonic, Toronto, Ontario).

### Statistical analysis

For cardiac function assessments, each group was individually analyzed using paired *t*-test to compare before and after treatments for each parameter. A percentage of change was calculated within each group for those parameters that were significantly different in the previous statistical test. Percentage of change from functional studies, morphometric quantitation from pathology, immunoreactive protein detection of 4HNE adducts, and all the enzymatic assays were analyzed using one-way ANOVA followed by Newman Keuls post-test (GraphPad Prism-4). A p value of less than 0.05 was considered a significant difference.
